# Impressions of the British Association Meeting

**Published:** 1911-09-16

**Authors:** 


### IMPRESSIONS OF THE BRITISH ASSOCIATION MEETING.

By a Medical Correspondent.

The annual meeting of the British Association has passed and the daily papers have provided their readers with summaries of papers read bearing upon various subjects of both speculative and practical interest. The most purely medical subject was perhaps the "progress of anæsthetics"; while the linking of medicine or physiology with danger entailed by some members of the community was illustrated by Dr. Leonard Hill's paper on the cause of "compressed air illness," and bacteriology with economics in the diseases of cultivated plants. Again the various papers which dealt with the mind, methods of investigation, and with education, appeal to all whose calling in life demands continuous study. To refer first to some of the last mentioned subjects, Professor Macdonald, in his presidential address before the physiological section, emphasised the difficulty of explaining the existence of the mind on physico-chemical phenomena alone, and said that he found it difficult to refrain from using the word "soul." The only suggestion that appears to be new in Professor Macdonald's address is his reference to the development of the eye in the embryo, and that light obviously plays no part in the process of its development. He indicated that just as light uses the eye which has been formed in the absence of light, so may something outside and independent of the brain use the brain as an instrument. Such a line of reasoning, needless to say, ignores the influence of heredity. An animal which through a long line of ancestors has acquired the colours of its desert surroundings will bear sandy-coloured children in our moist more or less green-coloured isle. Light may not directly have played any part in the development of the eye of a new-born infant, but it would require considerable boldness to assert that light has never been concerned in the development of the human eye.

Methods of investigation were in the main the basis of the presidential address of Professor H. H. Turner before the section of Mathematics and Physics. He controverted the view that scientific discovery necessarily follows working to establish the correctness of a theory. Professor Turner maintained such a statement as the following, made in 1886 by Sir G. H. Darwin, to be incorrect: "A mere catalogue of facts, however well arranged, has never led to any important scientific generalisation. For in any subjects the facts are so numerous and many-sided that they only lead us to a conclusion when they are marshalled by the light of some leading idea. A theory is then a necessity for the advance of science, and we may regard it as the branch of a living tree of which the facts are the nourishment." As an illustration of his (Professor Turner's) contention that a theory is unnecessary for the advance of knowledge, it was mentioned that Mr. W. W. Campbell, the Director of the Lick Observatory, decided to observe the line of sight velocities of as many stars as possible. A prolonged series of observations has shown that in comparison of the velocities with the spectra of various types of star, the older the star the quicker it moves. Possibly in venturing to criticise Professor Turner's contention, the old proverb "the exception proves the rule" might be mentioned. Now and again the aimless observer may hit upon some idea of value. His collections of facts may of themselves take shape. Speaking generally, some definite line upon which to work cannot but be an aid to intelligent observation. So fallible is the human mind, however, that there is always the danger that the observations may to some extent be injuriously influenced by the view held by the observer. Yet in spite of this the observer who cannot formulate a line of action for himself must in the majority of instances be devoid of one of the highest functions of the mind.

Bishop Welldon's remarks upon education contained much of considerable interest. One of his four main principles of education, though often held to be the aim of educationists, is worthy of being emphasised. This principle is "that the supreme object of education is to provide good citizens—citizens who, in Milton's stately language, will be able to 'perform justly, skilfully, and magnanimously all the offices, both public and private, of peace and war.'" The Bishop laid stress upon the need of the teacher for the study of the psychology and physiology of his art, and of a study of the individual character of his pupils. The teacher needed a practical insight to discover and encourage signs of genius or talent in a pupil, even when overlaid by many faults and failings. "There was no more humiliating reflection than that teachers had so frequently been blind to the promise of distinction in their pupils. Of the public schools especially it was only too true that they had been and in some degree still were the homes of the average and commonplace. They had applauded mediocrity if it conformed to the rules made by the masters for boys and the yet stricter rules made by boys for one another." Later praise was bestowed upon the civilising influence of elementary schools in some of the poorer districts of the large cities. It is customary to find fault with our educational methods, and from the practical standpoint they no doubt leave much to be desired. Our educational systems may be said to have passed through several experimental stages. Yet although fault may be found with our systems of education, both for the rich and for the poor, there can be no doubt that the Bishop was right when he praised the civilising influence of our elementary schools, as well as when he laid stress upon the necessity for realising that education is as much the teaching of the future citizen to acquire a sense of his responsibility towards others, as to develop to the full his own powers. The development of his powers, apart from a sense of responsibility to others, may make a Napoleon of him without adding to his own happiness or to the happiness of the world. In connection with education reference may be made to Dr. Abelson's mental tests for backward children. He maintained that the ordinary tests as to power of reading or of arithmetical ability are not good guides as to "practical intelligence." In this no doubt he is correct. Children of defective intelligence occasionally have considerable capacity in a certain direction. We know of a boy attending a school for "mentally defective children" who is said to be able to play from ear most tunes after once hearing them. It has never occurred to us to inquire whether the teachers are aware of his musical tastes, but it is unlikely that they would hear of it unless the boy's mother informed them. Dr. Abelson's tests include: (1) Tapping, (2) crossing out rings, (3) crossing out sets of dots, (4) immediate memory for sentences, (5) immediate memory for names, (6) immediate memory for commissions, (7) discrimination of length, (8) interpretation of pictures, (9) geometrical figures.

In his paper upon "Compressed air illness," Dr. Leonard Hill mentioned his views upon ventilation, namely that it is heat, moisture, and stillness of the air, not $CO_2$, which cause discomfort in ill-ventilated atmosphere. The writer of a leading article in one of the daily papers objects to this view, and considers the human nose to be a better judge of the cause of unpleasantness in a badly-ventilated room than the conductor of any number of experiments. It is not easy to see on what this line of reasoning is based. If the air around animals is poisonous according to the unpleasantness of its smell, then it is undesirable to keep some dogs, and a tame mouse must be a highly dangerous animal.

Sir Frederick Hewitt again pressed his views as to the need for reform in connection with the administration of chloroform. He asserted, what is impossible for any unprejudiced person to deny, that a considerable number of deaths during the administration of chloroform are preventible. In this connection it may be mentioned that we would like to see all the hearts of patients dying under chloroform anæsthesia submitted to an expert morbid anatomist or small committee of about three such anatomists for microscopical examination. From time to time we still see it recorded in the daily paper that death has resulted from "fatty degeneration of the heart." This we hold to be due to mistaken interpretation of post-mortem appearances.

